# Direct Ink Writing of Highly Conductive MXene Frames for Tunable Electromagnetic Interference Shielding and Electromagnetic Wave-Induced Thermochromism

**DOI:** 10.1007/s40820-021-00665-9

**Published:** 2021-06-22

**Authors:** Xinyu Wu, Tingxiang Tu, Yang Dai, Pingping Tang, Yu Zhang, Zhiming Deng, Lulu Li, Hao-Bin Zhang, Zhong-Zhen Yu

**Affiliations:** 1 State Key Laboratory of Organic-Inorganic Composites, College of Materials Science and Engineering, Beijing University of Chemical Technology, Beijing, 100029 People’s Republic of China; 2 Beijing Key Laboratory of Advanced Functional Polymer Composites, Beijing University of Chemical Technology, Beijing, 100029 People’s Republic of China; 3 Beijing Advanced Innovation Center for Soft Matter Science and Engineering, Beijing University of Chemical Technology, Beijing, 100029 People’s Republic of China

**Keywords:** MXene, Electromagnetic interference shielding, Direct ink writing, Electrical conductivity, Thermochromism

## Abstract

**Highlights:**

- 3D printing of MXene frames with tunable electromagnetic interference shielding efficiency is demonstrated.
- Highly conductive MXene frames are reinforced by cross-linking with aluminum ions.
- Electromagnetic wave is visualized by electromagnetic-thermochromic MXene patterns.

**Abstract:**

The highly integrated and miniaturized next-generation electronic products call for high-performance electromagnetic interference (EMI) shielding materials to assure the normal operation of their closely assembled components. However, the most current techniques are not adequate for the fabrication of shielding materials with programmable structure and controllable shielding efficiency. Herein, we demonstrate the direct ink writing of robust and highly conductive Ti_3_C_2_T_x_ MXene frames with customizable structures by using MXene/AlOOH inks for tunable EMI shielding and electromagnetic wave-induced thermochromism applications. The as-printed frames are reinforced by immersing in AlCl_3_/HCl solution to remove the electrically insulating AlOOH nanoparticles, as well as cross-link the MXene sheets and fuse the filament interfaces with aluminum ions. After freeze-drying, the resultant robust and porous MXene frames exhibit tunable EMI shielding efficiencies in the range of 25–80 dB with the highest electrical conductivity of 5323 S m^−1^. Furthermore, an electromagnetic wave-induced thermochromic MXene pattern is assembled by coating and curing with thermochromic polydimethylsiloxane on a printed MXene pattern, and its color can be changed from blue to red under the high-intensity electromagnetic irradiation. This work demonstrates a direct ink printing of customizable EMI frames and patterns for tuning EMI shielding efficiency and visualizing electromagnetic waves.
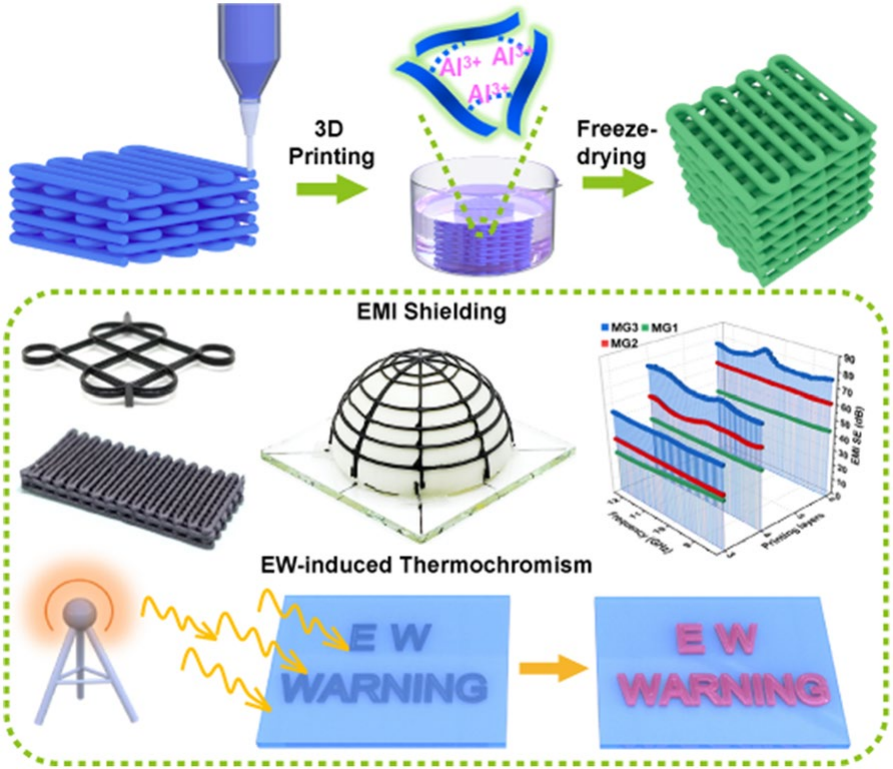

**Supplementary Information:**

The online version contains supplementary material available at 10.1007/s40820-021-00665-9.

## Introduction

Electromagnetic waves can propagate in the free space without any physical media, making it possible for cross-regional information communication. Although electromagnetic waves cannot be recognized with naked eyes, their existence relies on alternating electromagnetic fields, carrying a lot of electromagnetic energy to achieve long-distance information transmissions. To mitigate radiation and interference of electromagnetic waves to the human body or sophisticated electronic equipment, the electromagnetic energy should be reflected, absorbed, or dissipated to heat by electromagnetic radiation shielding materials [1].

Two-dimensional (2D) Ti_3_C_2_T_x_ MXene is firstly applied for electromagnetic interference (EMI) shielding in 2016 on the basis of its outstanding electrical conductivity, abundant surface hydrophilic functional groups, large specific surface area, and ease of process [2]. Till now, various MXene films [3, 4], wearable fabrics [5, 6], as well as multifunctional composites [7, 8] are developed with excellent EMI shielding performances. Three-dimensional (3D) EMI shielding materials are usually designed with layer-by-layer, porous, or isolated internal structures to attenuate electromagnetic waves effectively by their conductive or magnetic components [9, 10]. Porous structures can be constructed in the forms of aerogels or foams by random, directional [11, 12] and bidirectional freeze-drying [13, 14], dip-coating with foam templates [15], heterogeneous interface assembly [16, 17], and so on. Although the 3D MXene architectures prepared by the above approaches exhibit excellent EMI shielding performances, their shapes and internal structures are restricted by the forming molds and/or templates. It is currently difficult to prepare 3D MXene architectures with customized configurations.

In EMI shielding scenarios, conventional metal shields are gradually replaced by conformal shielding materials and compartmental shielding materials. To meet the requirement of shape-conformability, direct ink writing (DIW) is increasingly used as a cost-effective and easy-forming 3D printing technology for producing devices and equipment [18]. Its mild processing temperature and the advantage of integrating multiple materials make it a favorable choice for 3D composites [19, 20]. Recently, MXene-based architectures are fabricated on the basis of the DIW. Zhang et al*.* [21] designed complex patterns by using an additive-free aqueous ink of MXene with a concentration of ~ 36 mg mL^−1^. Yang et al*.* [22] printed MXene microlattice and rectangular hollow prism with large Ti_3_C_2_T_x_ flakes in a large concentration range of 15–50 mg mL^−1^. Orangi et al*.* [23] used superabsorbent polymer beads to absorb water in the Ti_3_C_2_T_x_ suspension and printed filaments with a predetermined morphology at the Ti_3_C_2_T_x_ concentration of 28.9 wt%. The printable MXene inks are also obtained by synergizing with different additives. For example, graphene oxide sheets and carbon nanotubes (CNTs) are incorporated with nitrogen-doped MXene (N-Ti_3_C_2_T_x_) for the fabrication of sodium-ion mixture capacitor electrodes [24]. Silver nanowires, MnO_2_ nanowires, and fullerenes are used as MXene ink additives for constructing stretchable supercapacitors [25]. To the best of our knowledge, there is still rare report on direct ink writing of conformable MXene-based EMI shielding materials.

As an extrusion printing technology, DIW facilitates the formation of a continuous conductance path inside an entire 3D architecture and meets the prerequisite of high electrical conductivity for EMI shielding [26, 27]. The flexibility of design makes it possible to prepare periodic unit structures on the basis of electromagnetic theories [28, 29]. Wang et al*.* [30] prepared 3D core–shell structured liquid metal/elastomer composites with adjustable shielding efficiency by varying printing layers. Other architectures, like split-ring resonator and wire strip with a negative index of refraction [31], Miura-Ori origami structure with the frequency-selective surface [32], and saw-tooth folding films with tunable electromagnetic shielding [33], can be constructed with the DIW technology. This technology is effective in bridging the gap between the conductive MXene sheets and the customized electromagnetic shielding devices.

Herein, we demonstrate a DIW route to construct highly conductive MXene-based frames with customizable architecture for EMI shielding and electromagnetic-thermochromism applications. The flowability of the MXene ink is coordinated by adding AlOOH nanoparticles to ensure its printable ability and the structural stability of printed architectures. The printed MXene/AlOOH (MA) filaments are stacked up layer by layer and closely overlapped with each other, constructing the internally connected 3D conductive frame. After freeze-drying, the electrically insulating AlOOH nanoparticles are removed by etching with hydrochloric acid to generate pores inside the printed architecture, while the aluminum ions cross-link the MXene sheets to enhance the structural stability of the printed architecture, enabling it to sustain ~ 3000 times load of its own weight. The gelled MXene frame (MG) exhibits a high electrical conductivity of 5,324 S m^−1^, and its EMI shielding effectiveness is over 80 dB in the X-band. Furthermore, the printed MG patterns are coated and cured with a synthesized thermochromic polymer to realize the visualization of electromagnetic waves by naked eyes on the basis of the electromagnetic energy and thermal energy conversion principle.

## Experimental

### Materials

Ti_3_AlC_2_ powder (400 mesh) and LiF (purity 99.99%) were purchased from Jilin 11 Technology (China) and Aladdin (China), respectively. AlOOH nanoparticles with an average particle size of 50 nm were provided by Beijing Dekedaojin (China). AlCl_3_·6H_2_O was supplied by Xilong Scientific Company. Polydimethylsiloxane (SYLGARD 184, PDMS) prepolymer and its curing agent were purchased from Dow Corning (USA) and used with a mass ratio of 10:1. Hydrochloric acid (HCl, 37 wt%), absolute ethanol, and chloroform (CHCl_3_) were all provided by Beijing Chemical Reagents (China). 10,12-pentacosadiynoicacid (PCDA, purity 98%) was purchased from Alfa Aesar Chemicals. PCDA was dissolved in absolute ethanol and filtrated to remove its polymerized component.

### Preparation of Few-Layer Ti_3_C_2_T_x_ MXene Sheets by Etching of Ti_3_AlC_2_ Flakes

LiF (8 g) was dissolved in 100 mL of HCl (9 M) in a polytetrafluoroethylene reactor to get a homogenous solution under stirring at room temperature. After Ti_3_AlC_2_ (5 g) was added into the LiF/HCl solution slowly, the etching reaction lasted for 42 h under stirring at a constant temperature of 35 °C. The etched product was washed with deionized water by vigorous shaking and centrifuged at 6000 rpm for 5 min to remove the acidic supernatant. The washing–centrifuging process was repeated until the pH of the dispersion was ~ 6. Subsequently, the etched product was diluted with deionized water and centrifugated at 1200 rpm for 20 min to remove the incompletely etched Ti_3_AlC_2_ or other bulky particles. The supernatant was collected and subjected to the separation procedure one more time. Finally, the supernatant was concentrated by centrifuging at a high speed of 10,000 rpm for 40 min to obtain a highly concentrated few-layer Ti_3_C_2_T_x_ paste with a solid content of approximately 18 wt% (~ 196 mg mL^−1^).

### Fabrication of Printable MXene/AlOOH Inks

Firstly, 2.5 g of the as-prepared MXene paste (18 wt%) was diluted with water to 9 wt% and transferred into a quartz mortar. Subsequently, AlOOH nanoparticles (100 mg) were gradually added into the mortar and the mixture was ground for at least 20 min. After the viscosity of the mixture increased significantly and there was no obvious aggregation of the nanoparticles, the pasty mixture was transferred to a syringe and centrifugated to remove bubbles with a planetary vacuum deaerator at a speed of 1000 rpm for 15 min, and designated as MA3 ink. Similarly, the inks with MXene/AlOOH mass ratios of 15:1, 10:1 were also prepared and designated as MA1and MA2, respectively. Table S1 listed the compositions and solid contents of the MA inks. For comparison, pure MXene ink with a solid content of 9 wt% was also prepared and denoted as pure MX ink.

### Construction of Frames by Direct Writing with Inks Followed by Etching and Cross-linking

The syringe was installed on a Jiachuangxing three-axis control direct writing machine (China). The printing pattern was designed by the Autodesk Computer Aided Design (Auto CAD) software and converted to the format of DXF and inputted into the direct writing machine to control the movement path of the syringe. On the basis of the flowability of the MA inks, the printing pressure was fixed at 15–20 psi (pounds per square inch), and the inner diameter of the needle was 410 μm. The skeletons printed with the MA1, MA2, and MA3 inks were designated as MA1, MA2, MA3, respectively. To remove the AlOOH component and cross-link the MXene sheets with aluminum ions, the as-printed self-supporting frames were immersed in a solution with 0.5 M AlCl_3_ and 0.1 M HCl for 2 h, washed with deionized water to remove the excess HCI and aluminum ions, and freeze-dried to obtain porous MXene-based frames. The gelled frames by using the inks with MXene/AlOOH mass ratios of 15:1, 10:1, and 9:2 were designated as MG1, MG2, and MG3, respectively.

### Synthesis of Thermochromic PDMS and Assembly with Printed MXene Frames

Thermochromic polydimethylsiloxane (t-PDMS) was prepared as follows. As the thermochromic component, PCDA was mixed with the prepolymer of PDMS, and polymerized under stirring with the UV light exposure for 1 min. The color change of the mixture from colorless to blue indicates the successful polymerization of PCDA in the prepolymer of PDMS. The polymerized PCDA was also called polydiacetylene (PDA). After the curing agent of PDMS was added, the blue mixture was coated on the surface of the as-printed MG pattern and cured at room temperature to obtain a t-PDMS-coated MG pattern.

### Characterization

Morphologies of the printed structure were observed with a Hitachi S4700 field emission scanning electron microscope (SEM). Few layered MXene sheets were evaluated by a Hitachi 7700 transmission electron microscope (TEM) and a Bruker multi-mode 8 atomic force microscope (AFM). Rheological behaviors of the inks were tested on an Anton Paar MCR302 rheometer equipped with a 40-mm tape parallel plate geometry (1.985°, cutoff gap of 50 µm) at 25 °C. Before the rheology tests, all inks were defoamed by centrifuging. Zeta potentials of MXene, AlCl_3_/HCl solution and the aqueous dispersion of AlOOH were measured with a Malvern Nano-ZS Zetasizer. MXene, MA, and MG were characterized by a Thermo Fisher ESCALAB 250 X-ray photoelectron spectroscope (XPS) and a Rigaku D/Max 2500 X-ray diffractometer (XRD). Electrical conductivities of the 3D printed skeletons were measured with a Four Probes RST-8 resistivity meter (China). The infrared images were obtained using a FLIR-H16 thermal imager (HikVision). EMI shielding performances were measured with a Keysight N5224B PNA series vector network analyzer (VNA) in the frequency range from 8.2 to 12.4 GHz. All the samples for the EMI shielding measurements were directly printed to conform the shape of the X-band waveguide cavity.

## Results and Discussion

### Robust MXene Frames Constructed by Direct Writing with Printable MXene/AlOOH Inks

**Fig. 1:**
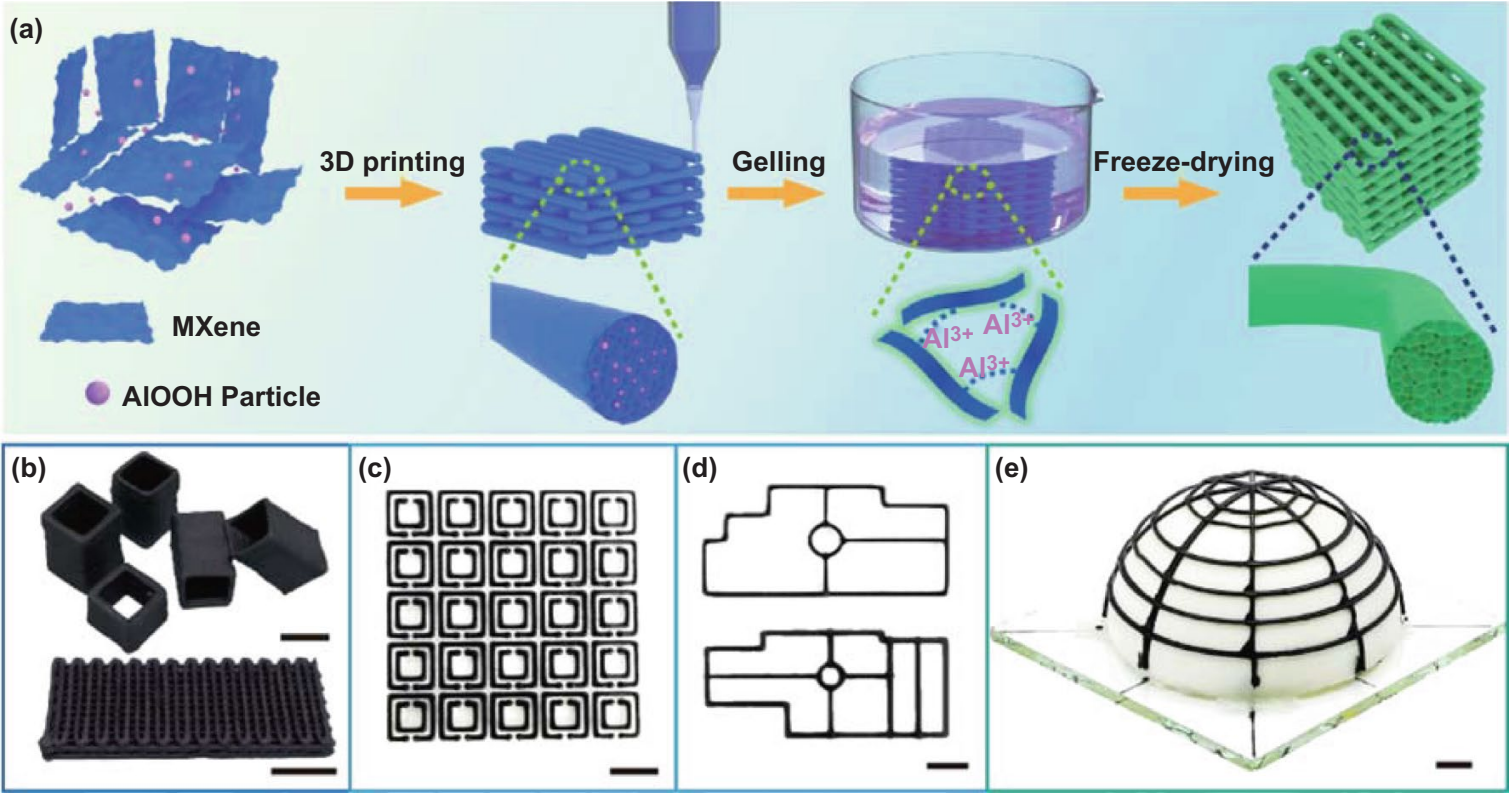
**a** Schematic of constructing a freestanding MG frame. **b** Printed MG cubic frame and “woodpile” microlattice. Printed MG with **c** double split rings and **d** the shape of EMI shields in electronic devices. **e** Adaptive hemisphere framework by direct ink printing. The scale bars in **Fig. 1 b-e** is 5 mm

Different from conventional EMI shielding materials, future shielding materials are required to satisfy higher integration density and higher design flexibility for more miniaturized and thinner electronic devices. Figure 1a shows the construction of customized porous MXene frames. First, AlOOH nanoparticles are added into the MXene paste to tune the printability and self-supporting performance, forming uniform MA inks. A pattern can be predesigned with the Auto CAD software, and constructed by a three-axis printing machine with the MA inks. The inks are extruded via a tiny nozzle by external air pressure and form filaments under the movements of the nozzle and the deposition bed. The filaments stack layer by layer with the nozzle lifting in the Z-axis direction to construct a complex 3D architecture (Fig. S1a). Subsequently, the printed MA frames are immersed in AlCl_3_/HCl solution for gelation, during which the AlOOH nanoparticles are removed on the basis of the reaction between AlOOH and HCl, and the MXene sheets are cross-linked by the aluminum ions derived from AlCl_3_ as well as the AlOOH, forming gelled MXene frames. Finally, freeze-drying is adopted to sublime the frozen water and maintain the porous structures of the MXene frames, leading to robust and lightweight MG frames.

Noteworthily, the versatility of DIW technique allows for free design and construction of regular architectures of sub-wavelength ranges according to specific radio frequencies. One of the prerequisites for printing 3D structures is that the printed filaments could maintain their shapes. For example, the printed ordered cuboids are composed of 20 vertically stacked monofilaments with clear borders and distinct morphology (Fig. 1b, upper). A sub-millimeter porous array with constant filament spacing can be printed readily (Fig. 1b, bottom and Fig. S1c), confirming the capability of the MA ink for clear and fine path designs [34]. By using the MA ink, "open resonant ring" and "commercial EMI shields" are printed as typically shielding architectures (Fig. 1c, d). Moreover, the printed hemispherical frame, "Chinese knots," and pentagrams with conformal sharp angles further confirm the adaptability of the ink for accurate printing of complex architectures (Figs. 1e and S1b).

As reported, few-layer MXene sheets are prepared by etching the MAX phase precursor with HCl/LiF solution, followed by delamination under vigorous hand-shaking. After etching, the XRD peak downward shifts from 9.5° to 6.3°, corresponding to the increase of interlayer spacing from 9.3 to 14.0 Å due to the intercalation of lithium ions and water molecules (Fig. S2a) [35]. Consistent with the greatly weakened (104) peak, the exfoliated MXene sheets are transparent with crumpled edges, indicating the ultrathin and flexible features (Fig. S2b). The MXene sheets are 1–4 μm in lateral sizes on the basis of the statistics of TEM images (Fig. S2c) and 3–5 nm in thickness (Fig. S2d), exhibiting a large aspect ratio and few structural defects.

Analogous to long-chain polymers, a dispersion of MXene sheets with a large aspect ratio usually exhibits high viscosity because of the sheet-to-sheet resistance under external shear forces [36]. The viscoelastic properties of an MXene dispersion can be adjusted by varying the MXene content to satisfy the different processing techniques [37], i.e., screen printing [38], spraying [39], and extrusion printing [40]. Generally, viscosities of the inks for directly writing should be in the range from 0.1 to 10^3^ Pa s [41]. Figure S3a shows apparent viscosities of pure MX ink and MA inks. The high viscosities of the inks at low shear rates indicate their solid-like state. Compared with MA2 ink, MA1 ink has a higher viscosity because of the higher content of MXene (Table S1). Although MA3 ink has the same overall solid content as MA2 ink, the former has a higher ratio of AlOOH nanoparticles. The increased viscosity of MA3 is attributed to the thickening effect of the AlOOH nanoparticles. The thickening effect of the AlOOH viscosifier is more obvious by comparing the viscosities of the MA3 ink and pure MX ink [42]. As the shear rate increases, the viscosity of all samples decreases rapidly, showing typical shear thinning characteristics. The effect of AlOOH nanoparticles on viscoelasticity is also confirmed by the oscillation amplitude tests (Fig. S3b). The storage modulus and loss modulus of the MA3 ink have an order of magnitude increase than that of pure MX ink. This indicates that the AlOOH nanoparticles increase rigidity and flow resistance of the ink, which would be beneficial for shape retention of the printed filaments [43]. All the MA inks have large plateau regions at initial shear stresses and their storage moduli are much higher than their loss moduli (G' > G''), presenting solid-like behaviors. When the shear stress approaches the yield stress, both the moduli drop sharply and viscous deformations occur (G' < G'') (Fig. S3c). Compared with MA1 ink, the modulus of MA3 ink increases more obviously, indicating that the addition of AlOOH nanoparticles enhances the resistance of ink to external force deformation. These results suggest that optimal viscoelastic properties of MA inks can satisfy the requirements for direct writing and the MG frames with different properties can be obtained by adjusting components of MA inks.

### Enhanced Mechanical and Electrical Performances of MXene Frames Reinforced by Cross-linking of MXene Sheets with Aluminum Ions

**Fig. 2:**
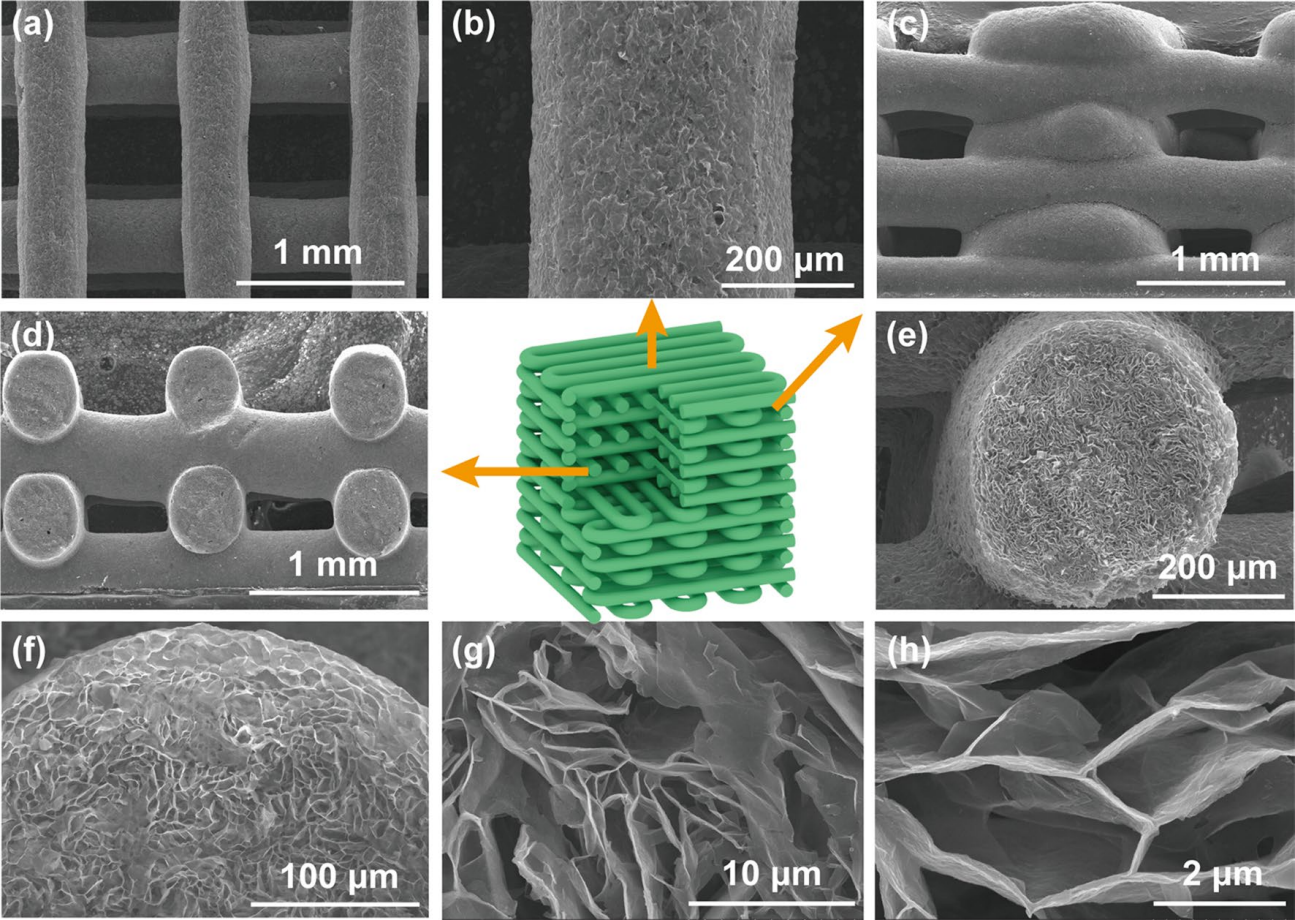
SEM images of porous MG2 with a wood-pile structure from different directions: **a, b** top view, **c** side view, and **d, e** cross-sectional view. **f–h** Cross-sectional SEM images of MG2 cross-linked by aluminum ions

To construct robust and porous MXene-based frames, the printed MA frames are immersed in HCl/AlCl_3_ solution to remove the electrically insulating AlOOH nanoparticles and to cross-link the MXene sheets by the aluminum ions. The dissolution of AlOOH with HCl generates numerous voids inside the filaments after freeze-drying, which would decrease the density of the printed frames. The *in situ* generated aluminum ions along with those of AlCl_3_ can cross-link the MXene sheets during the gelation process, reinforcing the filaments and enhancing joint between them. Figure 2 shows the morphology and microstructures of the wood-pile MG frames. Clearly, the individual filaments present well-defined uniform and distinct linear shapes without dislocation or fracture (Fig. 2a). Typically, the monofilament shows a diameter of 400 ± 30 μm, indicating the stable thixotropy and the self-supporting properties of the MA ink (Fig. 2b). The side-view (Fig. 2c) and cross-sectional view (Fig. 2d) SEM images show that the longitudinal filaments are arranged at equal intervals on the horizontal filaments. The crisscross filaments closely interconnect with each other to form continuous regular macroscopic architecture with jointed interfaces. In addition, the quadrilateral pores can be adjusted from millimeter to sub-millimeter by varying the filament spacing.

Moreover, the cross-section observation reveals the microporous feature of the MG frames (Fig. 2e, f). The fracture surface of filaments exhibits nearly circular appearances with densely packed micropores of 10 to 30 μm, which are attributed to the in situ subliming of the grown ice crystals during the freeze-drying process (Fig. 2f). Different from the random micropores for the MA frame (Fig. S4a, b), the "cabbage-like" microcellular structure of the cross-linked MG is more compact with better interconnection and continuity because of the gelation effect of aluminum ions (Fig. 2g). Furthermore, the abundant aluminum ions can interact with the dangling hydroxyl groups of MXene sheets to form a robust internal network (Fig. 2h). On the contrary, in the absence of the reinforcement of aluminum ions, the MXene sheets are weakly interconnected and disrupted by the growth of ice crystals, forming a random and fragile porous microstructure (Fig. S4c, d). MG1 and MG3 also exhibit "cabbage-like" microcellular structures due to the interaction of the aluminum ions with the MXene sheets (Fig. S5). The pores in the MG3 frame are more obvious than that of MG2 due to the lower density of MG3 (Table S2). A denser structure exists in the MG1 filaments because of its higher MXene content. The MXene sheets in all these frames are linked tightly by the aluminum ions to form the sinuous inner structures. Therefore, the introduced AlOOH nanoparticles not only improve the printability of the inks during the printing process, but also produce aluminum ions to *in situ* cross-link the MXene sheets, benefiting the structural stability of the printed frames even with low bulk densities (Table S2).

To figure out the reinforcement mechanism of aluminum ions, the printed MA3 frames are immersed in water, 0.1 M HCl solution, 0.5 M AlCl_3_ solution, and 0.5 M AlCl_3_/0.1 M HCl solution (Fig. S6). Clearly, the printed MA frames are readily collapsed after the immersion and washing processes in the absence of AlCl_3_. It means that the in situ generated aluminum ions by the etching of AlOOH are not sufficient for cross-linking all the MXene sheets to sustain the whole macroscopic 3D architecture. Besides, the weak interactions between the MXene sheets are not capable to resist the rehydration effect [44]. In contrast, the presence of AlCl_3_ provides sufficient aluminum ions to offer favorable interfacial interconnection and coalescence between the filaments (Fig. S6c) [45]. When the MA frame is immersed in the AlCl_3_/HCl solution, the printed frame keeps its customized structure because of the cross-linking effect with aluminum ions, and the removal of the AlOOH component is achieved simultaneously (Fig. S6d). These results confirm the reinforcement of printed architectures by the cross-linking with aluminum ions.

**Fig. 3:**
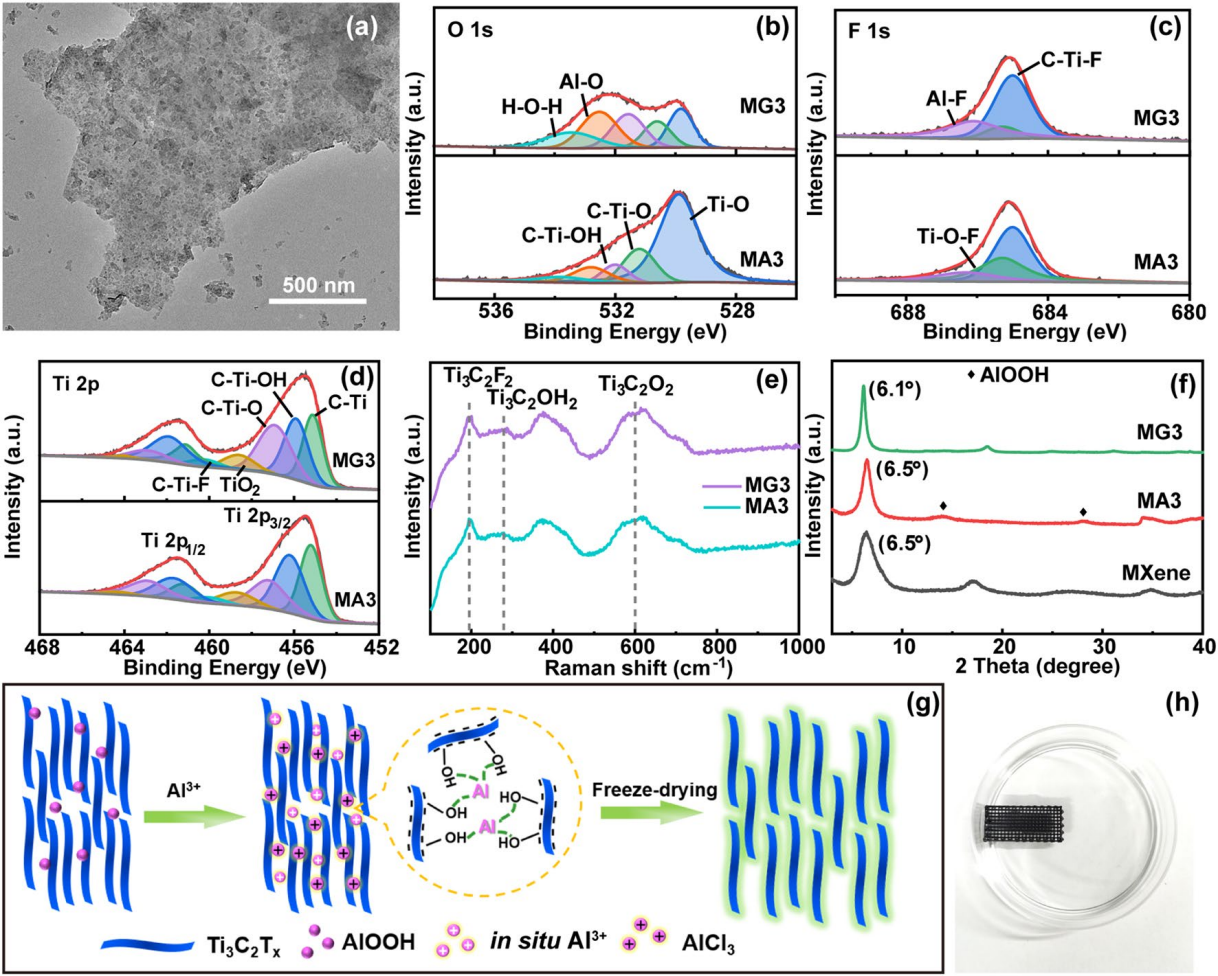
**a** TEM image of cross-linked MXene sheets extracted from a MG frame. **b** O 1 s, **c** F 1 s, and **d** Ti 2p XPS spectra of MA3 and MG3. **e** Raman spectra of MA3 and MG3. **f** XRD patterns of MXene, MA3, and MG3. **g** Aluminum ion-induced gelation of MXene sheets. **h** Intact MG3 frame after ultrasonication

As the MXene suspension presents negative zeta potential (− 27 mV) derived from the negative functional group of MXene sheets (Fig. S7a), the positive aluminum ions could link these sheets via electrical interactions. This phenomenon is further confirmed by the TEM image of the MXene sheets extracted from an MG3 frame by sonication (Fig. 3a). The evenly distributed AlCl_3_ crystals between the closely stacked MXene sheets indicate the strong interaction of aluminum ions with adjacent MXene sheets. The XPS spectra are shown in Fig. S7b. Consistently, as compared to MA3, MG3 presents an enhanced Al 2p peak and a stronger peak of Al-O bond at 532.5 eV (Figs. 3b and S7c), indicating the bonding of aluminum ions with oxygen-containing surface groups of MXene. In the F 1 s spectrum (Fig. 3c), the amplified Al-F peak is another evidence for the combination of aluminum ions with the negatively charged MXene sheets [46]. The Ti 2p XPS and Raman spectra of MA3 and MG3 do not have large changes, suggesting the maintaining of intrinsic properties of MXene during the gelation process [47] (Fig. 3d, e). The intercalation of aluminum ions between the MXene sheets is also verified by the downward shifted (002) peak of MG3 relative to that of MXene [48]. This phenomenon is also observed in the MG1 and MG2 frames due to the shift of (002) peaks in their XRD curves (Fig. S8a, b). The same elements and surface groups of MG1 and MG2 imply that the intrinsic properties of MXene are well kept after the cross-linking (Fig. S8c, d). In a word, as shown in Fig. 3g, the trivalent aluminum ions act as a linker to interconnect the MXene sheets and hence enhance their interfacial interaction.

The reinforcement of aluminum ions greatly improves the structural stability of the frame to resist external deformation. It can withstand a weight of almost 3000 times its own weight (Fig. S9a). Also, the aluminum ion-reinforced robust structure of MG3 enables it to resist ultrasonication for 2 min in water (Fig. S9b), as evidenced by the intact architecture of the MXene frame in water after the ultrasonication (Fig. 3h). The mechanical measurements further reveal the strengthening effect of the aluminum ions on the printed frames (Fig. S10). The compression strengths of MG frames are higher than those of their corresponding MA frames. Although there are plateau regions in the MA curves, the internal structures of MA frames are already destroyed when the strains reach their yielding points. The compression strength of MG3 is 186 kPa, much higher than that of MA3 (84 kPa). Similarly, MG2 has a higher compression strength of 241 kPa than that of MA2 (98 kPa). MG1 has the highest compression strength of 268 kPa, which is attributed to its highest density. All these results verify the reinforcement effect of the aluminum ions on the MG frames. The *in situ* generated aluminum ions from the AlOOH and the added aluminum ions from AlCl_3_ could well reinforce the interaction between MXene sheets inside the filaments and between the filaments, while maintaining the inherent conductive properties of MXene, which would ensure satisfactory EMI shielding performances of the MXene frames [49].

### Electrically Conductive and EMI Shielding Performances of Robust and Porous MXene Frames

**Fig. 4:**
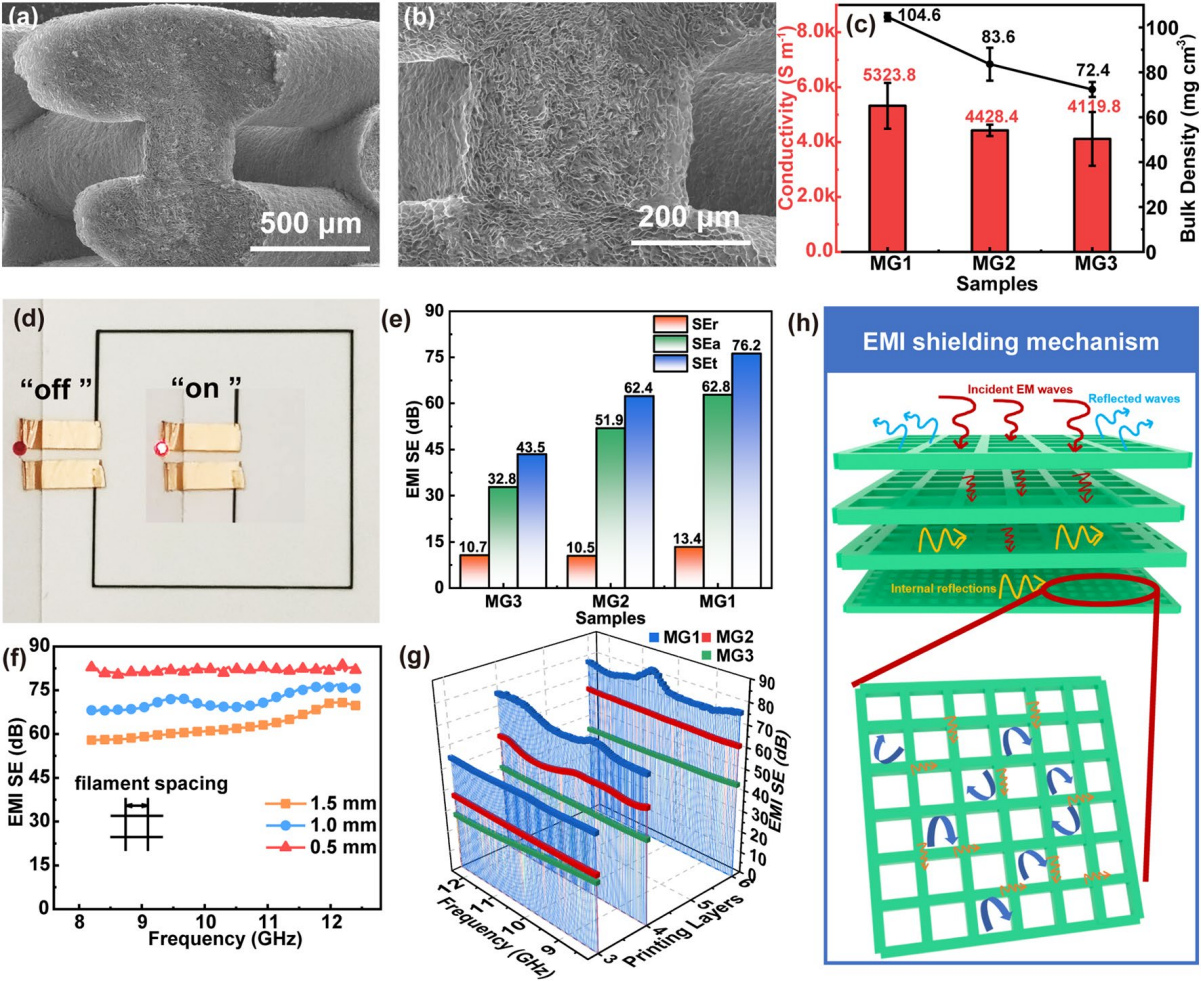
**a, b** SEM images of interfacial connection of MG2 frame. **c** Electrical conductivities and bulk densities of MG1, MG2, and MG3 frames with a constant filament spacing of 1 mm. **d** LED blub is lightened with an MG frame under an alternating electromagnetic field. **e** SEt, SEr, and SEa values of MG1, MG2 and MG3 frames with different filament constituents. The thickness is ~ 2 mm and the filament spacing is 1 mm. **f** EMI shielding performances of MG1 with different filament spacings. **g** Influences of printing layer on the EMI shielding performances of MG1, MG2, and MG3 frames. **h** EMI shielding mechanisms of MG frame

Thanks to the cross-linking of Al^3+^ ions, the MG frame fabricated by the versatile DIW method exhibits continuous interconnection between filaments with well jointed crisscross interfaces (Fig. 4a, b), endowing the lightweight and integrated hierarchical architectures with outstanding electrical conductivities. MG3 has an electrical conductivity of up to 4119 S m^−1^ with a bulk density of 71 mg cm^−3^, and the highest conductivity of 5,323 S m^−1^ is achieved for MG1 with a large MXene content (Fig. 4c). The high conductivity of the printed frame is well reflected by its electromagnetic induction experiment. For example, a LED bulb connected to the printed MG filament circuit can be lightened by the alternating magnetic field supplied by the induction cooker (Figs. 4d and S11, Video S1). Particularly, the generated alternating magnetic field can stimulate electrons of MXene sheets to freely transport through the conductive filaments, and lighten the LED light. The LED bulb can work normally if the magnetic field intensity is high enough. This result proves the good continuity and high conductivity of the printed MG filament circuit.

Furthermore, the versatile DIW technique can produce EMI shielding materials with predesigned structures for complex electronic packaging applications without post-treatment. For example, EMI shielding samples that are exactly adaptive to the waveguide cavity can be printed, and their shielding performances can be tuned finely by adjusting the printing layer number, filament constituents, and spacing. With a constant filament spacing of 1 mm and a thickness of ~ 2 mm, the overall EMI shielding performance is improved with increasing the MXene content in the filament (Fig. 4e), which is consistent with the increased tendency of electrical conductivity of the printed frames. MG3 delivers an average EMI SE value of 43.5 dB, which can block 99.99% of the incident electromagnetic waves. Among the explored samples, MG1 exhibits the highest EMI SE of 76.2 dB, superior to most of the 3D printing EMI shielding materials reported (Table S3). In addition, all these MXene frames show an absorption-dominant EMI shielding mechanism. By changing the filament spacing of MG1 from 1.5 to 1.0 mm, the average EMI shielding effectiveness (SE) increases from 62.7 to 71.5 dB (Fig. 4f). The normalized surface specific EMI SE of MG1-1 mm reaches 5044.1 dB cm^2^ g^–1^. Noticeably, further reduction in spacing to 0.5 mm affords an outstanding shielding performance of more than 80 dB over the entire X-band frequency range at a small thickness of 1.37 mm, and the normalized thickness specific SE is over 61.0 dB mm^−1^, exhibiting the advantage of the DIW fabrication of 3D electromagnetic shielding materials.

The frame thickness can be freely adjusted by controlling the printed layer number. The MG frames with 3, 4, and 6 layers are ~ 0.75, ~ 1.35, and ~ 2 mm in thickness, respectively. With the layer number changes, the EMI shielding performances can be tuned in the range of 25–76 dB (Fig. 4g). The excellent shielding performances can be ascribed to the high conductivity of the MG frame and its hierarchical porous structures with abundant interfaces [50].

The pore size of the printed frames also affects the EMI shielding performances [51]. As reported, the shielding performance in the X-band would be largely affected when the pore length is over 1.8 mm [52]. Fortunately, the square hole of the MG frame with a spacing of 1.5 mm is ~ 1.1 mm, smaller than one-tenth of the shortest wavelength of X-band electromagnetic waves and sufficient to block the free penetration of electromagnetic waves. Figure 4h shows the attenuation mechanism of electromagnetic waves by an MG frame. When the electromagnetic wave strikes the frame surface, a large part is reflected because of the high electron carrier density of the conductive MG frame. The incident wave is repeatedly scattered and diffracted inside the hierarchical porous structure, greatly extending the wave propagation path and leading to effective energy dissipation [53, 54]. In addition, the electromagnetic wave-activated electrons in the MG frame can migrate along the conductive filaments and jump across the interfaces or defects, generating micro-currents inside the filaments that would be beneficial for absorbing and dissipating the electromagnetic energy in the form of thermal energy [55, 56].

### Assembly of Thermochromic MG Patterns for Electromagnetic-Thermochromism and Electro-Thermochromism Applications

**Fig. 5:**
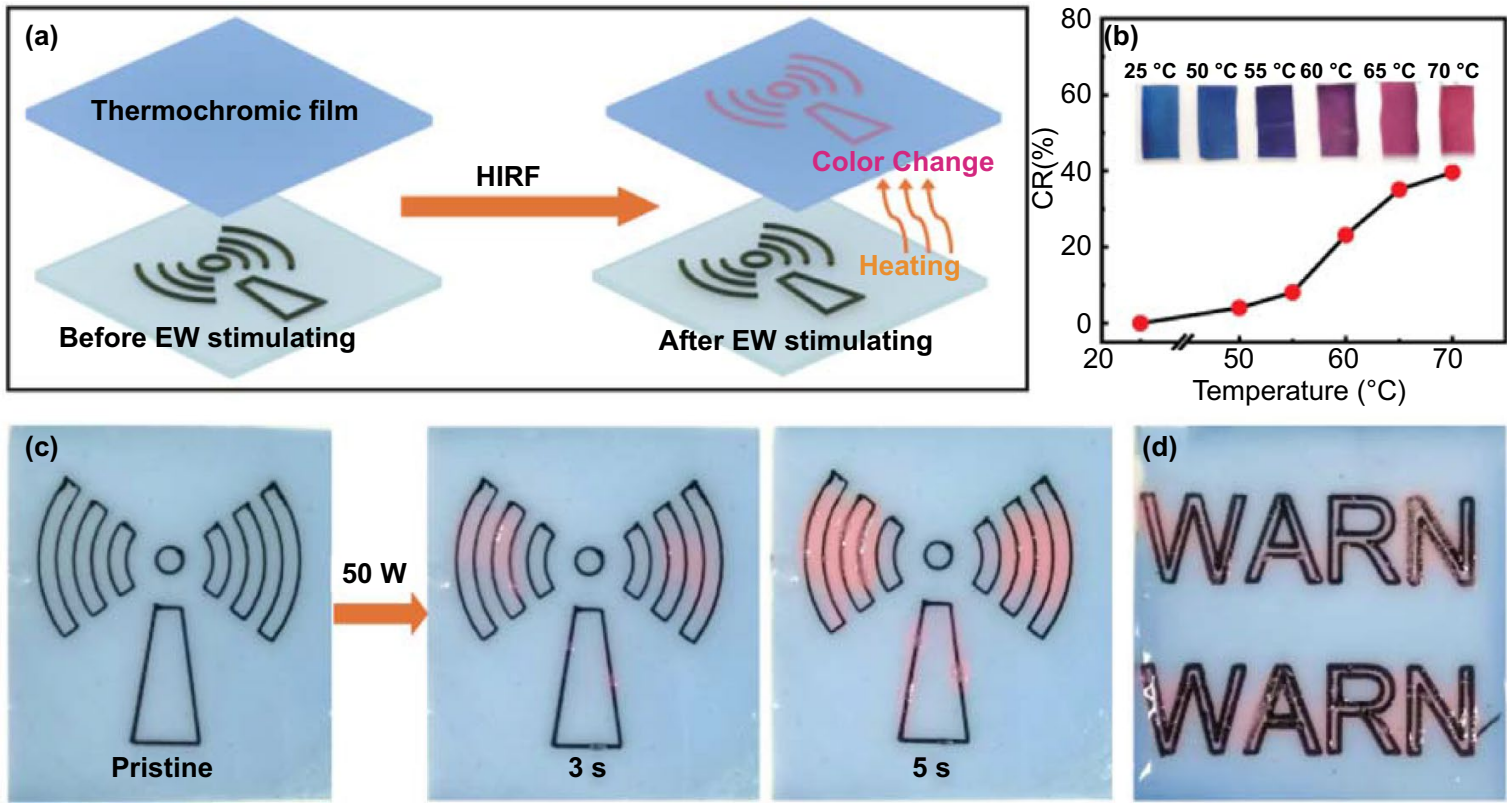
**a** Schematic of the fabrication of high-intensity radiated field (HIRF) responsive materials. **b** Colorimetric response (CR) values of t-PDMS from 25 to 70 °C. **c, d** Photographs of thermochromic MG3 patterns stimulated by electromagnetic waves of 2.45 GHz

Inspired by the conversion of electromagnetic energy to thermal energy, electromagnetic wave-induced thermochromic (EWT) patterns are developed by assembling the printed MG3 patterns with a thermochromic polymer of t-PDMS. Interestingly, high-intensity radiation field (HIRF) can be seen directly by our naked eyes because of the color change of the thermochromic MG patterns. This distinct color change derives from the conformational change of the PDA component of t-PDMS [57]. Stimulated by the heat generated by the irradiation of MG patterns with the strong alternating electromagnetic waves, the side-chain strains of PDA tend to release the mechanical strain generated by its polymerization. The side-chain distortion disturbs the p-orbital arrays to induce the color change from blue to red. The colorimetric response (CR) value calculated by the absorbance of t-PDMS in initial and final states can reach 40% (Figs. 5b and S13a) [58]. Apparently, t-PDMS exhibits a distinct color transition from blue to red with its temperature increases from 25 to 70 °C.

As shown in Fig. 5a, the "electromagnetic wave" pattern of MG3 is printed by the DIW technique using the MA3 ink on a glass base. After t-PDMS is coated and cured, the thermochromic MG3 is put in a microwave oven with a working frequency of 2.45 GHz, which can provide a strong alternating electromagnetic wave radiation field of 50 W (Fig. S12). Upon irradiation, the PDA directly contacted with MG3 quickly changes its color from blue to red within 3 s. The red color becomes more obvious when the thermochromic MG3 pattern is irradiated for another 2 s, indicating its temperature might be over 70 °C based on the color change of t-PDMS at different temperatures (Fig. 5b, c). However, the rest area of the t-PDMS that does not contact the MG3 pattern still maintains its original blue color. This phenomenon demonstrates that electromagnetic waves cannot cause the color change of t-PDMS itself. The thermal energy generated by interacting the alternating electromagnetic waves with the printed MG3 pattern is the origin of the color change of t-PDMS [59]. In addition, the filament interfaces or defects may cause charge accumulation and induce sparkle light, further confirming the severe interaction of MG3 with electromagnetic waves in the strong electromagnetic radiation field. In principle, the high-intensity alternating electromagnetic field enables charge carriers to migrate in a continuous printed pattern pathways [60]. The resultant induced current generates heat via vibration and migration of electrons, and thereby converts the invisible microwave to visible forms via the reaction-triggered color variation [61]. The shape of the MG pattern can also be designed as the word "WARN" (Fig. 5d), which could remind people to protect against high-intensity electromagnetic waves by the color change stimulated by strong alternating electromagnetic wave radiation fields. Based on these results, it is reasonable to design more sensitive EWT composites that can detect low-intensity electromagnetic waves by synthesizing low-temperature responsive thermochromic polymers. The visualization of electromagnetic waves is promising for potential warning of daily electromagnetic radiation, high-intensity electromagnetic pulse, and other related fields.

**Fig. 6:**
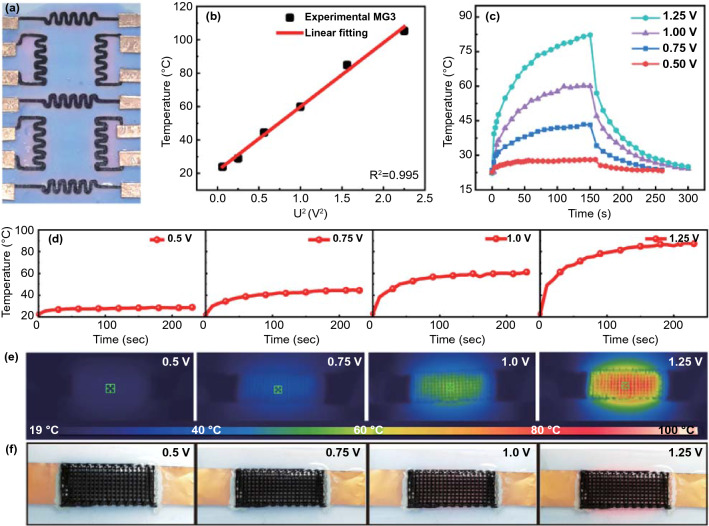
Electric thermal performances of printed MG3. **a** Digital photograph of printed thermochromic pattern. **b** Relationship between saturation temperature and U^2^ for MG3 pattern. **c** Time-dependent surface temperatures under different voltages. **d** Joule heating performances of MG3 pattern, **e** infrared thermal imaging photographs, and **f** color changes of electro-thermochromic MG3 pattern under related voltages

In addition to the electromagnetic wave-induced thermochromism, the electro-thermochromism of the thermochromic MG patterns is also proved by settling in a direct current source [62]. The temperature-sensitive color variation can act as an apparently visible indicator to in situ reflect the real temperature and voltage. Figure 6a shows the physical image of an electro-thermochromic MG3 pattern. The color change from blue to red directly indicates the excessively high temperature and applied voltage. With the MG3 frame as the Joule heater, the good linear relationship between the saturation temperature and the square of the voltage conforms well with the fitted curve, making it possible to finely control the target temperature with the applied voltage (Fig. 6b) [63]. Interestingly, the high conductivity of the printed MG3 pattern even affords a high temperature of 105 °C under a low voltage of only 1.5 V.

Figure 6c shows the temperature dependence of the MG3 pattern under different voltages, exhibiting good heating–cooling ability. As the applied voltage increases, the heating rate and the saturation temperature are greatly elevated. At 1.25 V, the temperature rises from 23 to 83 °C within 160 s and then decreases to room temperature after 140 s once the voltage is turned off, presenting a good heating–cooling recovery ability. A stable temperature of 42 °C can be kept at only 0.75 V, satisfying the requirement for keeping warm or relieving pain and stiffness of muscles. Figure 6d shows the time-dependent surface temperatures under different voltages, further confirming the low-voltage joule heating performance of the printed MG circuit. The temperature increase can be seen from the infrared thermal imager, while it also can be figured out by the color change from blue to red of the MG3 pattern (Fig. 6e, f). The electro-thermochromic ability of thermochromic MG patterns provides a way to recognize excessive temperature by naked eyes. Moreover, the MG3 pattern also has excellent long-term stability and recyclability, as evidenced by the stable temperature of 60 °C under 1 V (Fig. S13b). All these results verify that the thermochromic MG patterns can also be utilized to assemble smart electric heating devices with color change when the temperature is excessively high.

## Conclusions

Robust and highly conductive MXene frames with customized structures are fabricated by a direct ink writing technique with printable MXene/AlOOH inks for tunable electromagnetic interference shielding and electromagnetic-thermochromic applications. The 3D frames with predesigned structures are readily printed by combining MXene with AlOOH nanoparticles to form printable inks and then immersed in HCl/AlCl_3_ mixture to remove the electrically insulating AlOOH nanoparticles and reinforce the MXene architecture by cross-linking of MXene sheets with aluminum ions inside and between the printed filaments. After freeze-drying to subliming the frozen water, the resultant porous and robust MG frame can sustain a weight almost 3000 times of its weight, also exhibits a high electrical conductivity of 5,323 S m^−1^ and a satisfactory EMI shielding efficiency of 80 dB in the whole X-band. The surface specific SE achieves 5,044.1 dB cm^2^ g^–1^ when MG has a thickness of 1.35 mm and a bulk density of 0.105 g cm^−3^. The EMI shielding efficiency can be tuned in the range of 25 to 80 dB by adjusting the printing parameters. Furthermore, by assembling the printed MG patterns with a thermochromic t-PDMS synthesized, the resulting thermochromic MG patterns exhibit a distinct color change from blue to red on the basis of electromagnetic-thermochromism and electro-thermochromism, which is driven by the heat converted from electromagnetic energy and electric energy. The assembled thermochromic MXene-based patterns are promising in the fields of electromagnetic anti-counterfeit labels, strong electromagnetic radiation warning, electromagnetically or electrically converted thermal management equipment.

## Supplementary Information

Below is the link to the electronic supplementary material.

Digital photographs of printed patterns, and printing process; XRD patterns of MAX and MXene; TEM and AFM images of MXene; histogram of lateral sizes of MXene; viscosity of MA1 ink; plots of storage modulus and loss modulus of MA1 ink; SEM images of MA filaments and architectures; Zeta potentials; XPS spectra; digital photographs of MA2 frame; stable MG2 frame; light MG frame floats on water; photographs of MG3 under compression test; compression stress–strain curves; facility and schematic of an electromagnetic wave induction experiment; color change of thermochromic MG3; UV−vis spectra; temperature–time curve; relationship of MG2 between saturation temperature versus U^2^; electric thermal performances, surface temperatures, and temperature stability of MG2; calculation of colorimetric responses; preparation parameters for frames and their densities. (PDF 1749 KB)

Supplementary file2 (MP4 32012 KB)
