# Supplementary material for: Direct Ink Writing of Highly Conductive MXene Frames for Tunable Electromagnetic Interference Shielding and Electromagnetic Wave-Induced Thermochromism
Source: Nanomicro Lett. 2021 Jun 22;13:148. doi: 10.1007/s40820-021-00665-9 (PMC8219826; doi:10.1007/s40820-021-00665-9)
Supplement: Supplementary file 1 — Digital photographs of printed patterns, and printing process; XRD patterns of MAX and MXene; TEM and AFM images of MXene; histogram of lateral sizes of MXene; viscosity of MA1 ink; plots of storage modulus and loss modulus of MA1 ink; SEM images of MA filaments and architectures; Zeta potentials; XPS spectra; digital photographs of MA2 frame; stable MG2 frame; light MG frame floats on water; photographs of MG3 under compression test; compression stress–strain curves; facility and schematic of an electromagnetic wave induction experiment; color change of thermochromic MG3; UV−vis spectra; temperature–time curve; relationship of MG2 between saturation temperature versus U2; electric thermal performances, surface temperatures, and temperature stability of MG2; calculation of colorimetric responses; preparation parameters for frames and their densities. (PDF 1749 KB) [file 40820_2021_665_MOESM1_ESM.pdf]

Supporting Information

Direct Ink Writing of Highly Conductive MXene Frames for Tunable Electromagnetic Interference Shielding and Electromagnetic Wave-Induced Thermochromism

Xinyu Wu[1,2], Tingxiang Tu[1], Yang Dai[1], Pingping Tang[1], Yu Zhang[1], Zhiming Deng[2], Lulu Li[2], Hao-Bin Zhang[1,2*], Zhong-Zhen Yu[2,3*]

[1] State Key Laboratory of Organic-Inorganic Composites, College of Materials Science and Engineering, Beijing University of Chemical Technology, Beijing 100029, China

[2] Beijing Key Laboratory of Advanced Functional Polymer Composites, Beijing University of Chemical Technology, Beijing 100029, China

[3] Beijing Advanced Innovation Center for Soft Matter Science and Engineering, Beijing University of Chemical Technology, Beijing 100029, China

*Corresponding authors.

E-mails: zhanghaobin@mail.buct.edu.cn (H.-B. Zhang); yuzz@mail.buct.edu.cn (Z.-Z. Yu)

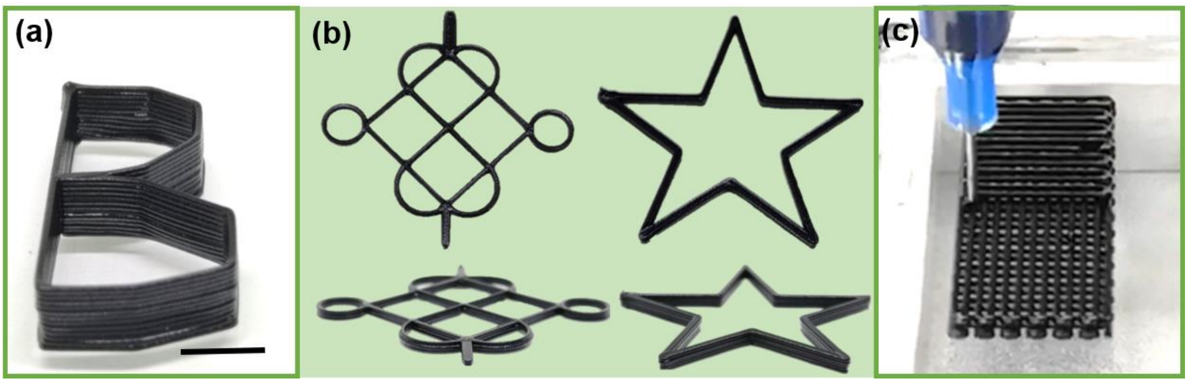

Fig S1. Digital photos of **a** printed letter "B" with 15 layers. **b** Top- and side-views of printed patterns of "Chinese knot" and a "pentagram". **c** Printing process for a wood-piled frame.

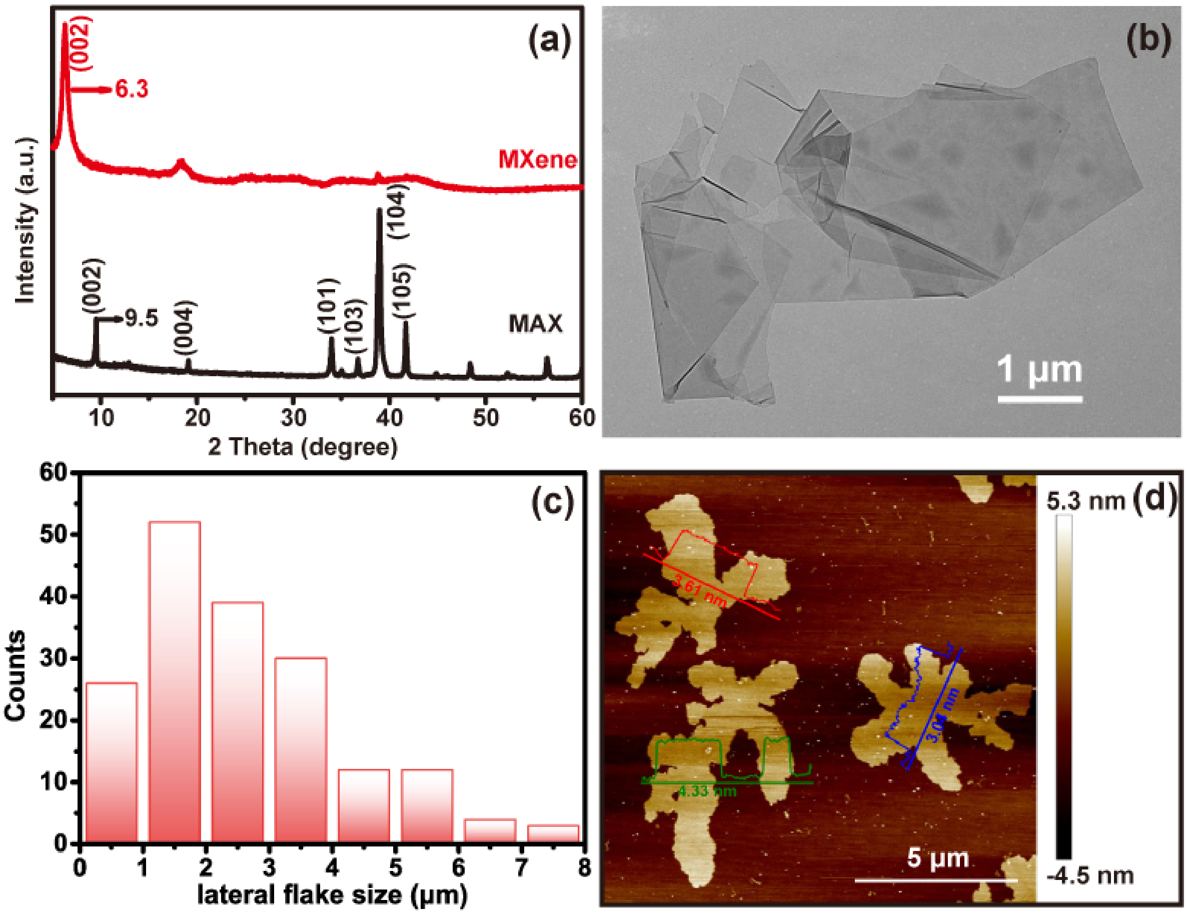

Fig S2. a XRD patterns of MAX and few-layered $Ti_3C_2T_x$ MXene. **b** TEM image of MXene. **c** Histogram of lateral sizes of MXene sheets on the basis of TEM images of diluted MXene suspension. **d** AFM image of MXene sheets.

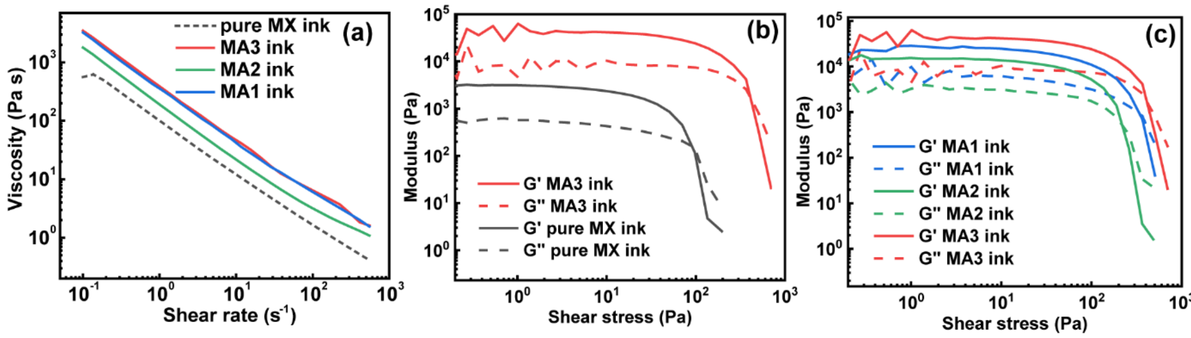

Fig S3. **a** Shear viscosity of pure MX ink with a MXene content of 9 wt%, MA1 ink, MA2 ink, and MA3 ink. **b** The addition of AlOOH nanoparticles increases the storage modulus (G') and loss modulus (G") greatly. **c** Plots of storage modulus (G') and loss modulus (G") of MA1, MA2, MA3 inks as a function of shear stress.

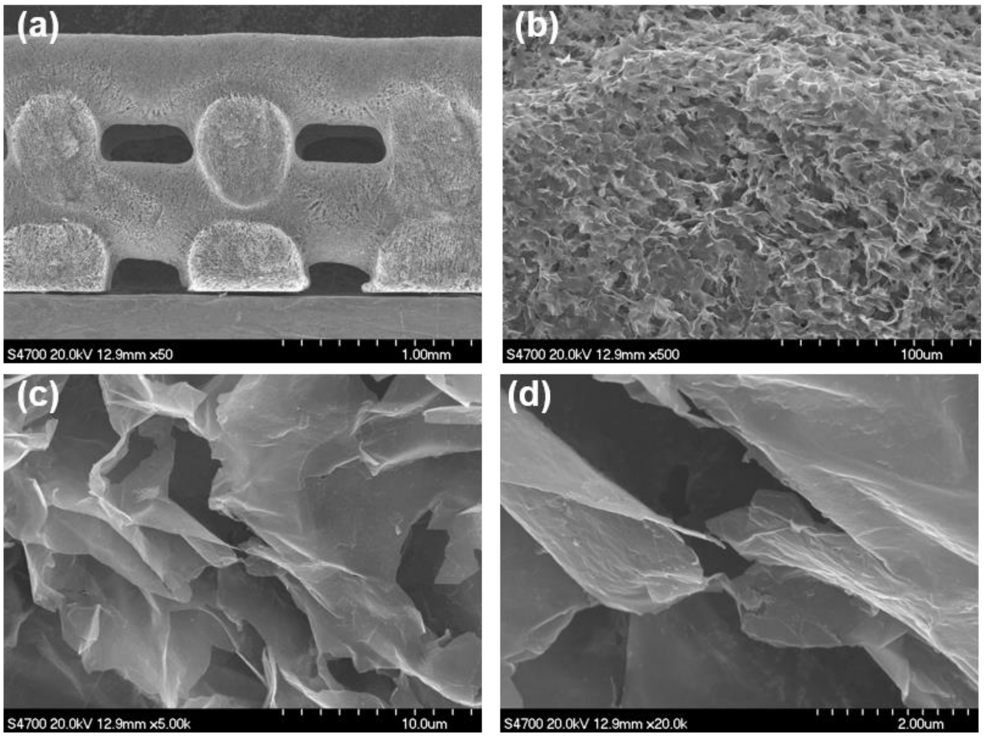

Fig S4. SEM images of an MA architecture without crosslinking. **a** Cross-sectional view of the fracture surface of filaments. **b** High-magnification SEM image of a filament. **c, d** Detailed microstructure of the MA frame without crosslinking.

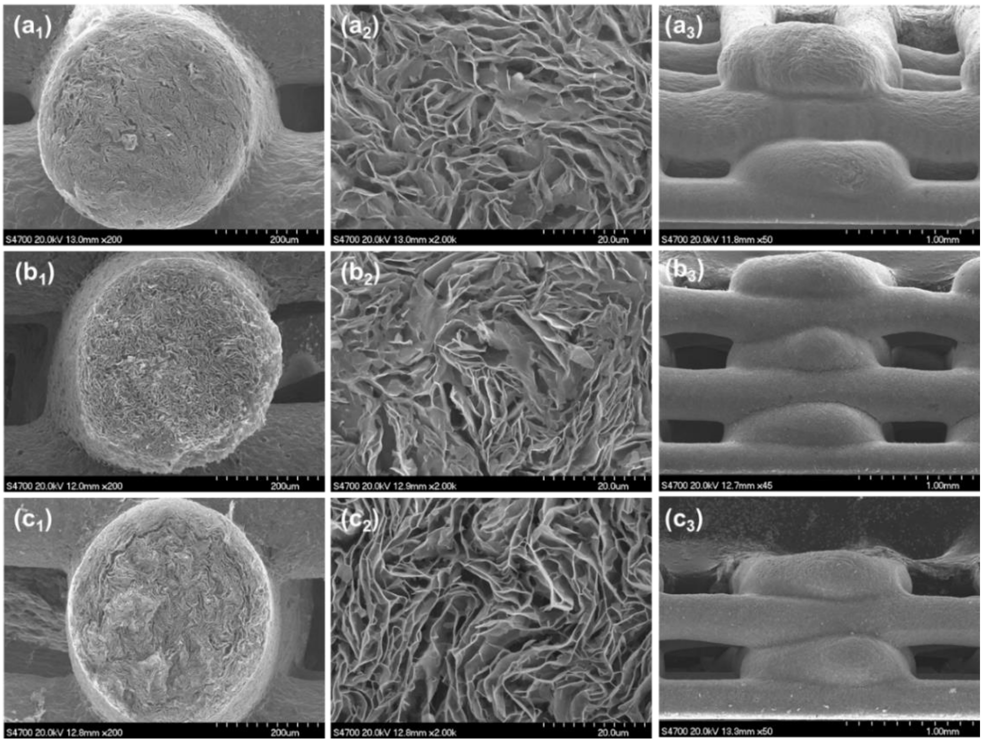

Fig S5. SEM images Cross-sectional of **a_1, a_2** MG1, **b_1, b_2** MG2, **c_1, c_2** MG3 frames and side view of **a_3** MG1, **b_3** MG2, **c_3** MG3 frames.

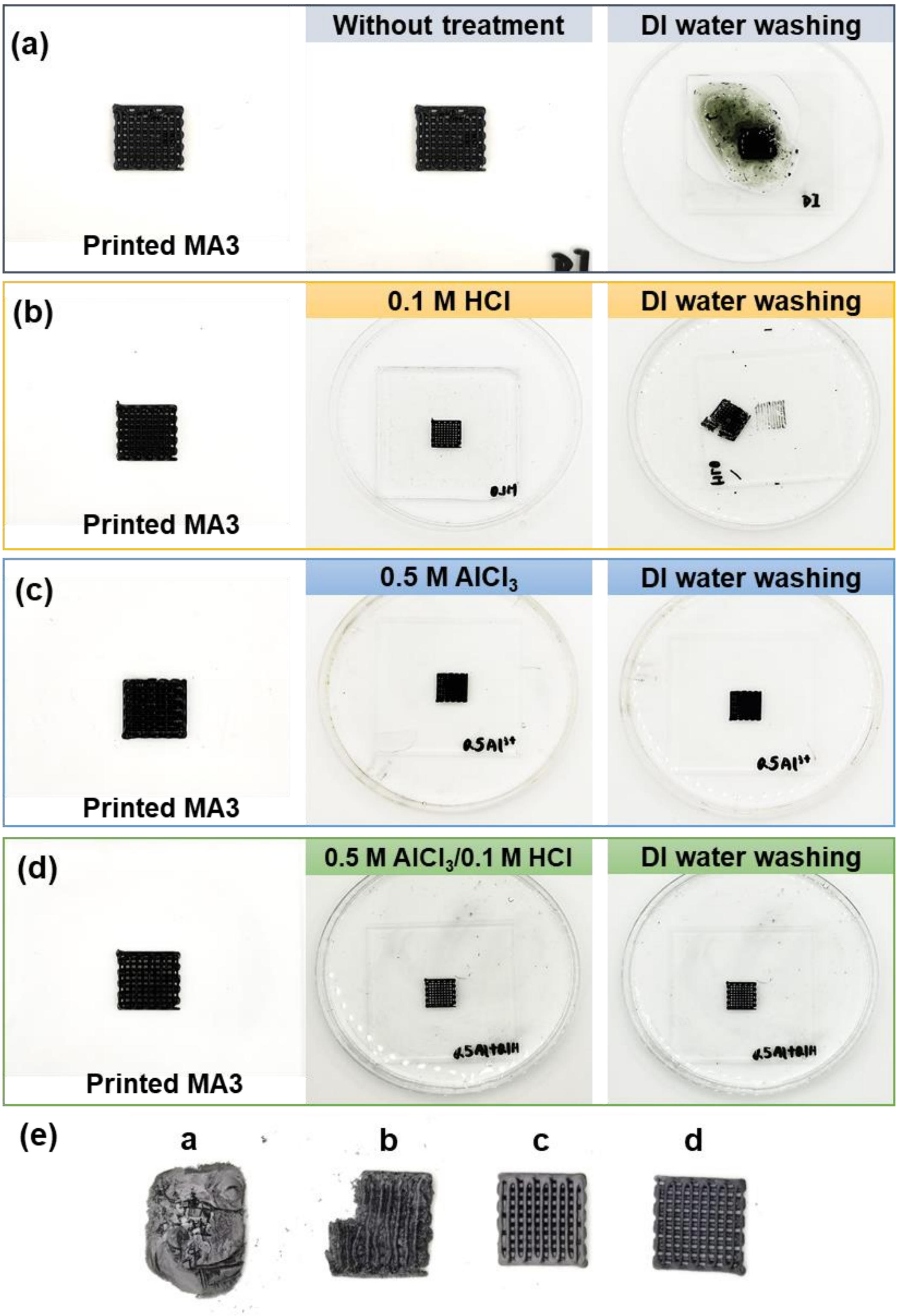

Fig S6. a MA3 frame is immersed in water. **b** MA3 frame is immersed in 0.1 M HCl solution. **c** MA3 frame is immersed in 0.5 M $AlCl_3$ solution. **d** MA3 frame is immersed in 0.5 M $AlCl_3$/0.1 M HCl solution. **e** Corresponding samples after the immersion treatments and washing with water.

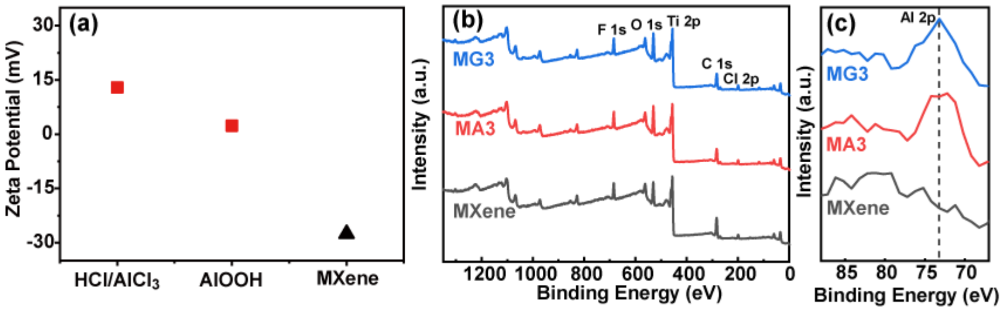

Fig S7. **a** Zeta potentials of HCl/$AlCl_3$ solution, AlOOH suspension, and MXene suspension. **b** XPS spectra of MXene, MA3, and MG3. **c** Al 2p curves of MXene, MA3, and MG3.

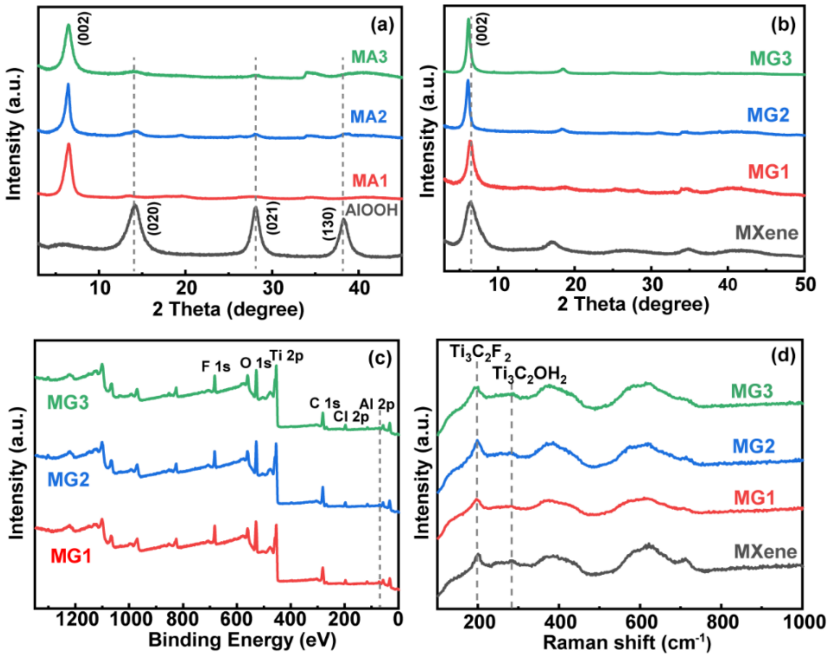

Fig S8 **a** XRD patterns of AlOOH, MA2 and MA3. **b** XRD patterns of MXene, MG1, MG2 and MG3. **c** XPS spectra of MG1, MG2 and MG3. **d** Raman spectra of MXene, MG1, MG2 and MG3.

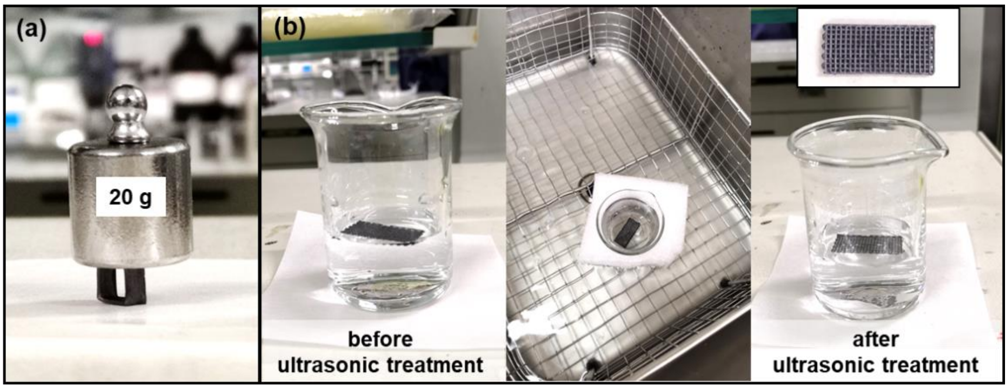

Fig S9. **a** Stable MG3 frame (6.8 mg) sustains a weight of 20 g. **b** Light MG frame floats on water, and there is no obvious structural change after ultrasonication at 240 W for 2 min.

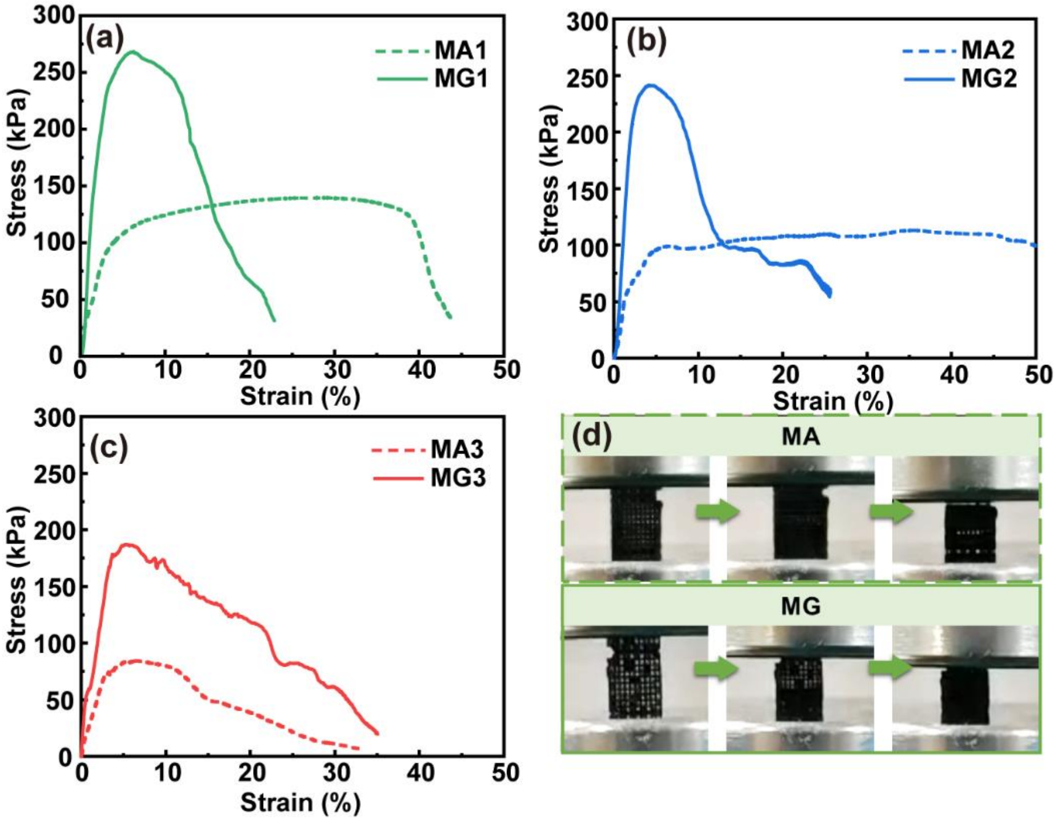

Fig S10. Compression stress-strain curves of **a** MA1 and MG1, **b** MA2 and MG3 and **c** MG3 and MA3. **d** The digital photographs of MA and MG frames during compressing tests.

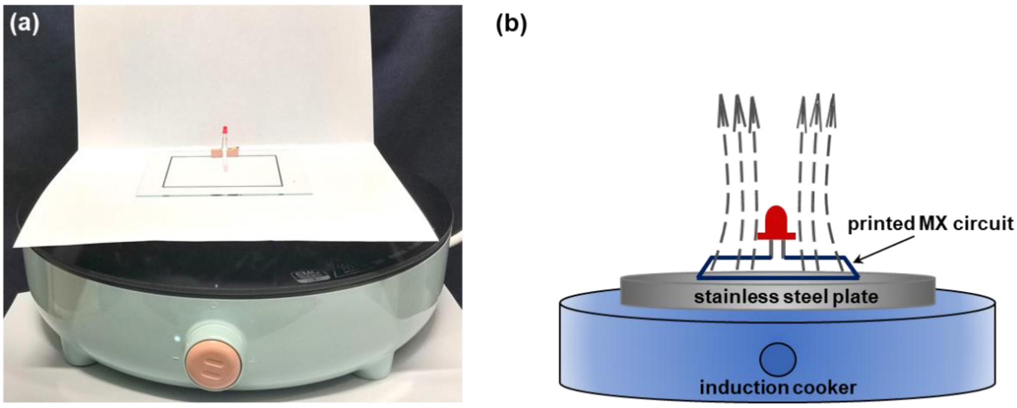

Fig S11. **a** Facility and **b** schematic of an electromagnetic wave induction experiment. Base on the electromagnetic induction principle, the induction cooker provides a high-frequency alternating magnetic field, which could generate eddy currents in the stainless-steel plate. The magnetic field generated by the change of eddy currents passes through the conductive MXene printed circuit, resulting in the transmission of electrons between MXene sheets. Thus, current can be formed inside the printed MXene filaments to lighten a LED bulb.

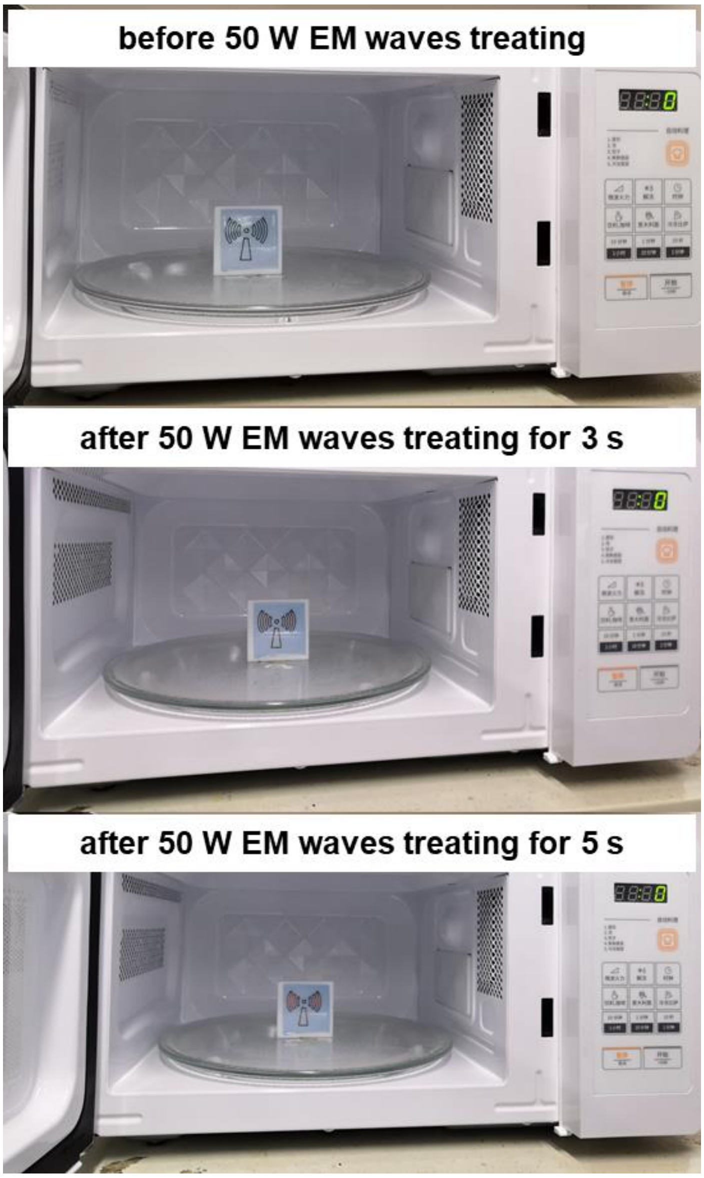

Fig S12. Color change of thermochromic MG3 under irradiation for different times in a microwave oven with a working frequency of 2.45 GHz**.**

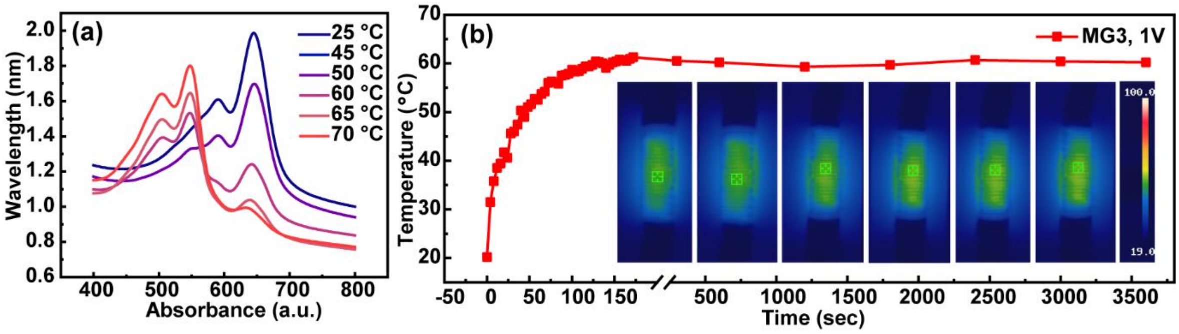

Fig S13. a UV−vis spectra of t-PDMS under different temperatures. **b** Temperature-time curve of MG3 at a constant voltage of 1 V.

The calculation of colorimetric response (CR) value:

$CR = (PB_0 - PB_f) / PB_0 \times 100\ \%$

$PB = A_{645\ nm} / (A_{645\ nm} + A_{548\ nm})$

The subscripts 0 and f represent the initial and final states of t-PDMS; *A* represents the absorption peaks of UV-vis spectra at 645 nm and 548 nm.

Table S1. The components of MA inks.

Ink names	The solid contents of MXene (wt%)	The solid contents of AlOOH (wt%)	The total solid contents of MA inks (wt%)
MA1	15	1	16
MA2	10	1	11
MA3	9	2	11

Table S2. The preparation parameters of frames and their densities.

Samples	Gelation	Spacing between Filaments (mm)	Bulk density (mg cm^{-3})
**MA3**	No	1.0	96.3 ± 13.2
**MA2**	No	1.0	97.8 ± 4.2
**MA1**	No	1.0	115.3 ± 0.5
**MG3**	Yes	1.0	72.4 ± 3.3
**MG2**	Yes	1.0	83.6 ± 7.3
**MG1**	Yes	0.5	162.4 ± 6.8
**MG1**	Yes	1.0	104.6 ± 1.8
**MG1**	Yes	1.5	89.4 ± 5.0

Table S3. Preparation methods and EMI shielding performance of typical 3D materials

Materials	Methods	Electrical conductivity (S/m)	EMI SE [8.2~12.4 GHz] (dB)	Thickness (mm)	Density (g/cm^3)	SE/d (dB/mm)	SSE/t ($dB/cm^2.g$)	Ref.
MXene aerogel	A	2200	75	——	0.02	——	——	[S1]
MXene/SA aerogel	A	2211	70.5	2	0.021	35.25	16785.7	[S2]
MXene aerogels	B	——	48.5	1	0.0055	48.50	88182.0	[S3]
MXene/graphene foam	C	1250	50.7	3	0.0072	16.9	23472.2	[S4]
Cu nanowires/ graphene	C	2083	52.5	9.5	0.0165	5.53	3349.3	[S5]
SiC foam	D	23	24.2	3	0.87	8.07	92.7	[S6]
Carbon Foam	D	240	51.2	2	0.154	25.60	1662.3	[S7]
CNT/PLA	FDM	20	67.5	2.0	——	33.8	——	[S8]
CNT/PLA	FDM	5000	30	0.7	0.4	42.9	214.3	[S9]
Ag @ CNF/ PLA	FDM	2.1×10^5	46	~0.35	~2	131.43	657.1	[S10]
Liquid metal/elastomer	DIW	~ 3.4×10^6 (EGaIn)	82	9.5	~ 6.4 (EGaIn)	8.63	——	[S11]
Polyethylene/ graphene	FDM	——	29	2.05	0.44	2.05	318.0	[S12]
MG1-0.5 mm#	DIW	5323	81.8	1.34	0.162	61.04	3768.2	This work
MG1-1.0 mm#	DIW	5323	71.5	1.35	0.105	52.96	5044.1	
MG1-1.5 mm#	DIW	5323	62.7	1.38	0.089	45.43	5105.0	
MG1##	DIW	5323	76.2	2	0.105	38.10	3628.6	
MG2##	DIW	4428	62.4	2	0.084	31.20	3714.3	
MG3##	DIW	4119	43.5	2	0.072	21.75	3020.8	

A: Directional freeze-casting; B: Bidirectional freeze-casting; C: freeze-casting; D: Template-based method; # These MG frames are 4 layers with different filament spacings; ## These MG frames are 6 layers with the filament spacing of 1mm.

References

[S1]. R. Bian, G. He, W. Zhi, S. Xiang, T. Wang, D. Cai, Ultralight MXene-based Aerogels with High Electromagnetic Interference Shielding Performance. J. Mater. Chem. C **7** (3), 474-478 (2019). https://doi.org/10.1039/c8tc04795b

[S2]. X. Wu, B. Han, H.B. Zhang, X. Xie, T. Tu, Y. Zhang, Y. Dai, R. Yang, Z.Z. Yu, Compressible, Durable and Conductive Polydimethylsiloxane-Coated MXene Foams for

High-performance Electromagnetic Interference Shielding. Chem. Eng. J. **381**, 122622 (2020). https://doi.org/10.1016/j.cej.2019.122622

[S3]. M. Han, X. Yin, K. Hantanasirisakul, X. Li, A. Iqbal, C.B. Hatter, B. Anasori, C.M. Koo, T. Torita, Y. Soda, L. Zhang, L. Cheng, Y. Gogotsi, Anisotropic MXene Aerogels with a Mechanically Tunable Ratio of Electromagnetic Wave Reflection to Absorption. Adv Optical Mater. **7** (10), 1900267 (2019). https://doi.org/10.1002/adom.201900267

[S4]. Z. Fan, D. Wang, Y. Yuan, Y. Wang, Z. Cheng, Y. Liu, Z. Xie, A Lightweight and Conductive MXene/graphene Hybrid Foam for Superior Electromagnetic Interference Shielding. Chem. Eng. J. **381**, 122696 (2020). https://doi.org/10.1016/j.cej.2019.122696

[S5]. S. Wu, M. Zou, Z. Li, D. Chen, H. Zhang, Y. Yuan, Y. Pei, A. Cao, Robust and Stable Cu Nanowire@Graphene Core-Shell Aerogels for Ultraeffective Electromagnetic Interference Shielding. Small **14**(23), 1800634 (2018). https://doi.org/10.1002/smll.201800634

[S6]. C. Liang, Z. Wang, L. Wu, X. Zhang, H. Wang, Z. Wang, Light and Strong Hierarchical Porous SiC Foam for Efficient Electromagnetic Interference Shielding and Thermal Insulation at Elevated Temperatures. ACS Appl. Mater. Interfaces **9** (35), 29950-29957 (2017). https://doi.org/10.1021/acsami.7b07735

[S7]. L. Zhang, M. Liu, S. Roy, E.K. Chu, K. Y. See, X. Hu, Phthalonitrile-Based Carbon Foam with High Specific Mechanical Strength and Superior Electromagnetic Interference Shielding Performance. ACS Appl. Mater. Interfaces **8** (11), 7422-7430 (2016). https://doi.org/10.1021/acsami.5b12072

[S8]. Y. Wang, Z.W. Fan, H. Zhang, J. Guo, D.X. Yan, S. Wang, K. Dai, Z.M. Li, 3D-Printing of Segregated Carbon Nanotube/Polylactic acid Composite with Enhanced

Electromagnetic Interference Shielding and Mechanical Performance. Mater. Des. **197**, 109222 (2021). https://doi.org/10.1016/j.matdes.2020.109222

[S9]. K. Chizari, M. Arjmand, Z. Liu, U. Sundararaj, D. Therriault, Three-Dimensional Printing of Highly Conductive Polymer Nanocomposites for EMI Shielding Applications. Mater. Today Commun. **11**, 112-118 (2017). https://doi.org/10.1016/j.mtcomm.2017.02.006

[S10]. H. Wei, X. Cauchy, I. O. Navas, Y. Abderrafai, K. Chizari, U. Sundararaj, Y. Liu, J. Leng, D. Therriault, Direct 3D Printing of Hybrid Nanofiber-Based Nanocomposites for Highly Conductive and Shape Memory Applications. ACS Appl. Mater. Interfaces **11** (27), 24523-24532 (2019). https://doi.org/10.1021/acsami.9b04245

[S11]. Z. Wang, J. Ren, R. Liu, X. Sun, D. Huang, W. Xu, J. Jiang, K. Ma, Y. Liu, Three Dimensional Core-shell Structured Liquid Metal/Elastomer Composite via Coaxial Direct Ink Writing for Electromagnetic Interference Shielding. Compos. Part A: Appl. S. **136**, 105957 (2020). https://doi.org/10.1016/j.compositesa.2020.105957

[S12]. J. Jing, Y. Xiong, S. Shi, H. Pei, Y. Chen, P. Lambin, Facile Fabrication of Lightweight Porous FDM-Printed Polyethylene/Graphene Nanocomposites with Enhanced Interfacial Strength for Electromagnetic Interference Shielding. Compos. Sci. Technol. **207**, 108732 (2021). https://doi.org/10.1016/j.compscitech.2021.108732
